# Supplementary material for: Causal Relationship between Aging and Anorexia Nervosa: A White-Matter-Microstructure-Mediated Mendelian Randomization Analysis
Source: Biomedicines. 2024 Aug 16;12(8):1874. doi: 10.3390/biomedicines12081874 (PMC11351342; doi:10.3390/biomedicines12081874)
Supplement: Supplementary file 1 [file biomedicines-12-01874-s001.zip › Supplementary Figure/Figure S4/Singe SNP plot of MR analysis for TL to ubm-b-1754.pdf]

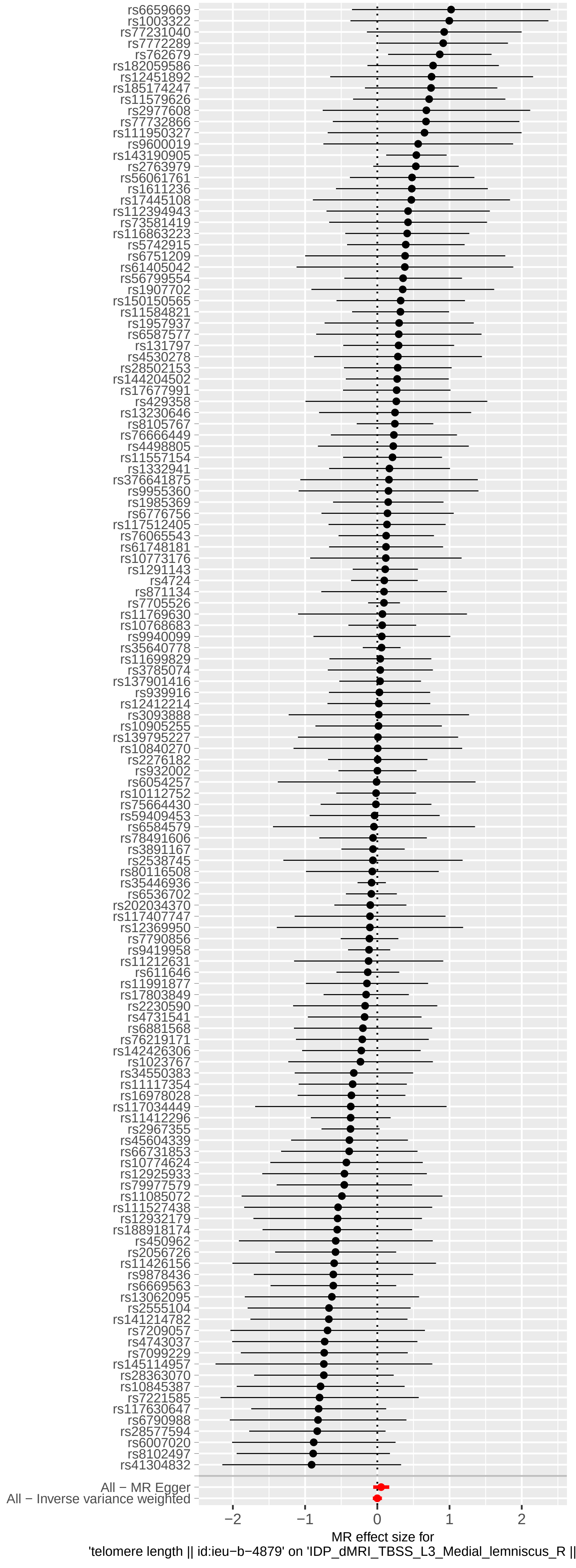
rs6659669
rs1003322
rs77231040
rs7772289
rs762679
rs182059586
rs12451892
rs185174247
rs11579626
rs2977608
rs77732866
rs111950327
rs9600019
rs143190905
rs2763979
rs56061761
rs1611236
rs17445108
rs112394943
rs73581419
rs116863223
rs5742915
rs6751209
rs61405042
rs56799554
rs1907702
rs150150565
rs11584821
rs1957937
rs6587577
rs131797
rs4530278
rs28502153
rs144204502
rs17677991
rs429358
rs13230646
rs8105767
rs76666449
rs4498805
rs11557154
rs1332941
rs376641875
rs9955360
rs1985369
rs6776756
rs117512405
rs76065543
rs61748181
rs10773176
rs1291143
rs4724
rs871134
rs7705526
rs11769630
rs10768683
rs9940099
rs35640778
rs11699829
rs3785074
rs137901416
rs939916
rs12412214
rs3093888
rs10905255
rs139795227
rs10840270
rs2276182
rs932002
rs6054257
rs10112752
rs75664430
rs59409453
rs6584579
rs78491606
rs3891167
rs2538745
rs80116508
rs35446936
rs6536702
rs202034370
rs117407747
rs12369950
rs7790856
rs9419958
rs11212631
rs611646
rs11991877
rs17803849
rs2230590
rs4731541
rs6881568
rs76219171
rs142426306
rs1023767
rs34550383
rs11117354
rs16978028
rs117034449
rs11412296
rs2967355
rs45604339
rs66731853
rs10774624
rs12925933
rs79977579
rs11085072
rs111527438
rs12932179
rs188918174
rs450962
rs2056726
rs11426156
rs9878436
rs6669563
rs13062095
rs2555104
rs141214782
rs7209057
rs4743037
rs7099229
rs145114957
rs28363070
rs10845387
rs7221585
rs117630647
rs6790988
rs28577594
rs6007020
rs8102497
rs41304832
All − MR Egger
All − Inverse variance weighted
−2
−1
0
1
2
MR effect size for
'telomere length || id:ieu−b−4879' on 'IDP_dMRI_TBSS_L3_Medial_lemniscus_R ||
